# The Impact of Partnerships and Information Sharing on Corporate Sustainable Performance: A Mediation Model Moderated by Government Support

**DOI:** 10.3389/fpsyg.2022.942279

**Published:** 2022-07-11

**Authors:** Longfei Yue, Meng Ye, Qing Chen

**Affiliations:** ^1^College of Physical Education, Sichuan University, Chengdu, China; ^2^Business School, Sichuan University, Chengdu, China

**Keywords:** partnerships, information sharing, sustainable performance, government support, cooperation, trust

## Abstract

Based on the theory of strategic alliances and social networks, this article empirically studies the relationship between partnership, information sharing, and sustainable performance through a questionnaire survey of Chinese sports equipment manufacturers. The findings show that partnerships have a positive impact on sustainable performance; that information sharing plays a role in mediating the relationships between trust, cooperation, and sustainable performance; and that government support can positively impact the effect of partnerships on sustainable performance. Through empirical research, this article proves the mechanism of the impact of partnership on alliance performance, further expands the theoretical basis for enterprises’ establishment of strategic alliances, and has important enlightening significance for enterprises within alliances aiming to rationally use the networks inside and outside their alliances to obtain knowledge and resources and improve their sustainable performance.

## Introduction

The global pandemic of COVID-19 has cast a shadow over the development prospects of the world sports industry (Ratten et al., 2021). The epidemic has brought serious threats to life and health, and has caused tremendous psychological shock. More people realize that in the face of the epidemic, medical care can only cure the symptoms, and only physical fitness can cure the root cause (Leguizamo et al., 2021). Psychological changes are manifested in behavioral improvements in sports participation and more emphasis on individual sports behaviors. The change of concept will give birth to a new consumption pattern of sports products. For example, people pay more attention to the consumption of personal sporting goods, such as sportswear, fitness equipment, wearable fitness equipment, etc. Many scholars predict that in the post-epidemic era, the sports market will usher in retaliatory consumption and the sports industry will recover (Schnitzer et al., 2020).

In the past, the sports industry continued to maintain rapid growth and still has a large room for growth in the future. It is of great significance to adhere to sustainable development in the growth process of the sports industry (Huang and Chen, 2022). The sporting goods manufacturing industry has strong industrial relevance. Its upstream and downstream industrial chain is not only related to agricultural production such as cotton in the primary industry; it is also related to industrial production such as textiles, rubber, steel, and construction in the secondary industry; it is even related to modern services such as warehousing, logistics, sales, and packaging in the tertiary industry. This also means that the production process and production method of the sporting goods manufacturing industry must consume a large amount of natural resources, energy and industrial water, and at the same time generate a large amount of solid waste and greenhouse gases. In addition, due to the mismatch between supply and demand, a large number of primary sporting goods are often left unused or disposed of at low prices, resulting in a waste of resources. To promote the sustainable development of the sporting goods manufacturing industry, it is impossible to rely on a single sporting goods manufacturing enterprise. Alliances must be formed in the overall sporting goods manufacturing industry chain. The enterprise alliance balances the economic interests, environmental benefits and social responsibilities of enterprises internally, and externally drives the upstream and downstream industrial chains to explore and jointly promote green manufacturing and green production.

Early business managers were influenced by the “economic man” hypothesis, believing that the driving force of the survival and development of enterprises was the continuous acquisition of economic benefits and maximization of shareholder profits; indeed, it was not until the birth of stakeholder theory and social responsibility theory that entrepreneurs began to focus on the balance between economic and social interests (Roscoe et al., 2019; Shahzad et al., 2020). With the gradual awakening of environmental awareness, the question of whether and how enterprises assume environmental responsibilities to achieve green development and sustainable development has begun to become a topic of great interest for scholars and entrepreneurs (Abbas and Sagsan, 2019; Tian et al., 2022). There are multiple coexisting worldviews of corporate sustainability, but the most dominant worldview is focused on the business case for sustainability, a position anchored in the weak sustainability paradigm (Baumgartner, 2014; Baumgartner and Rauter, 2017) believe that in addition to being responsible to shareholders, companies should also be environmentally responsible, focusing on the sustainable enterprise development from both economic and environmental perspectives. Enterprises actively carrying out environmental management can directly promote their performance, and environmental management can help enterprises establish a sustainable and stable competitive advantage (Montiel and Delgado-Ceballos, 2014). Many businesses are increasingly using strategic partnerships to manage corporate environmental agendas. Alliance partnerships are of great significance to the sustainable development of enterprises (Sadovnikova and Pujari, 2017; Russo and Schena, 2021).

As the study of strategic alliances began to deepen, Nakamura (2005) found that the prevalence of strategic alliances is accompanied by high failure rates and instability. The main reason for the rupture of strategic alliances is the difficulty of maintaining relationships between partners (van Beers and Zand, 2014; Dyer et al., 2018). The study of the relationship between partnerships and alliance performance has gradually attracted the attention of scholars. However, the results of such empirical research are inconsistent: some scholars believe that there is a positive relationship between partnerships and the development of strategic alliances (Hottenrott and Lopes-Bento, 2016; Jiao et al., 2019; Iwami, 2021). From the perspective of social networks, some scholars have discussed the influence of alliance network structure characteristics on alliance performance and found that interenterprise network connections can have an important impact on alliance performance (Kamal et al., 2021). Other scholars believe that there is an inverse relationship between these two factors (McEvily et al., 2003). The most critical reason for these conflicting results is that most of the existing research on the relationship between partnership and alliance performance focuses on only the direct impact of partnership on alliance performance, ignoring the intermediate effect between the two.

In contrast to general alliance situations, alliance partnerships, information sharing, and government support have important impacts on the sustainable development of alliances. This article argues that information sharing plays an intermediary role in the impact of alliance partnerships on alliances’ sustainable performance and further argues that this intermediated relationship is impacted by government support. Finally, by examining the adjusted mediation model, this article analyzes the process mechanism of the impact of alliance partnerships on the sustainable performance of alliances, providing a reference for alliance management practice.

**FIGURE 1:**
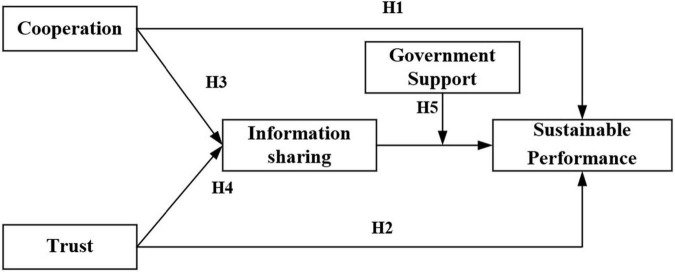
Proposed model and hypotheses.

The article is structured as follows. The second part presents the relevant research on partnerships, information sharing, government support, and sustainable alliance performance, makes research hypotheses, and constructs the theoretical model shown in Figure 1. The third part provides the method. The fourth part presents the data analysis and a discussion. The last part concludes the research.

## Theoretical Model and Hypotheses

### Alliance Partnerships and Sustainable Performance

The literature in the field of strategic management suggests that relationship quality refers to the “degree of relationship bonding” and reflects the nature of strategic relationships. The relationship between enterprises affects the individual behavior of the members of the organization, which in turn affects the performance of the enterprise. Literature in the field of supply chain management shows that high-quality relationships between node companies and upstream and downstream partners can help improve the efficiency of the entire supply chain and promote long-term and stable transactions between the two parties. Huntley (2006) pointed out that the measurement of relationship quality should take into account both relationship behavioral factors (such as trust, cooperation) and environmental factors. The main characteristics of interalliance partnerships are trust, cooperation and a high level of information sharing. Factors such as an inability to accurately define the degree of partnership, an uneven distribution of benefits and risks, a lack of sufficient trust in alliance partners, and the possibility of potential partner benefits being less considerable than their external risks can lead to the failure of partnerships (Martin et al., 2016). Alliance partners need to exhibit qualities such as mutual trust, concern for alliance development, an understanding of partner needs, and positive responses to alliance actions (Wu et al., 2014). Trust is essential to initiate, establish, and maintain social relationships (Balliet and Van Lange, 2013). Based on the perspective of current research, this article believes that the measurement indicators of enterprise alliance partnerships mainly include cooperation and trust.

#### Cooperation and Sustainable Performance

Many studies have contended that cooperation in strategic alliances can effectively improve the finance-based performance of participants, such as cost and revenue, and their non-finance-based performance, such as customer service and market commercialization (Luo, 2008; Zhang et al., 2010). Other studies have discussed interfirm collaborations with links to both financial and non-financial performance for partners. Financial performance includes cost efficiency and return on investment, and non-financial performance may range widely, including factors such as reduced uncertainty through vertical integration, access to complementary resources, and risk avoidance *via* coinvestment with partners (Angel, 2002; Aarstad et al., 2015; Tran et al., 2021).

Firms must have sufficient capabilities and knowledge to respond to and develop solutions for current dynamic environmental demands. Therefore, cooperation with partners has become crucial to enabling firms to acquire basic knowledge, conduct competitive research, respond to new demands, and gain access to networks while increasing their reputations and thus improving their positions in the market (Arroyave et al., 2020). Furthermore, cooperation with partners has turned in an opportunity for firms to obtain funding for research projects conducted by administrative bodies; on the other hand, it has also turned into an opportunity to implement long-term technological strategies to make the most of opportunities offered to them by the public R&D system (Bayona-Saez et al., 2002). Choi and Choi (2021) found that vertical R&D cooperation positively affected overall industry performance, especially on service and marketing performance.

Hence, considering that cooperation with partners increases firm’s information sharing and the development of their sustainable performance and that we expect a positive relationship between information sharing and firm performance, we propose direct and indirect effects of cooperation and performance. Cooperation increases a firm’s knowledge base and its image and reputation. This reputation also improves if a firm is environmentally sound and developing sustainable performance (Moreno-Mondejar et al., 2020). Therefore, we propose the following:

*H1: Cooperation has a significant positive impact on an alliance’s sustainable performance.*

#### Trust and Sustainable Alliance Performance

Management scholars have studied trust from multiple perspectives, including those of the social exchange literature (Colquitt et al., 2012, 2013; Su et al., 2020), economics, marketing and operations, and supply chain management (Poppo et al., 2016). Trust is regarded as a key variable in developing and maintaining relationships. The effect of trust on enhancing performance in interorganizational relationships has been widely recognized in existing literature. Trust is a necessary condition for resource sharing among enterprises, a representative social capital factor, and a topic that is generally a concern of social capital theory (Chen et al., 2014; Lu et al., 2016). Trust can be defined as a firm’s expectation that its partners will perform a particular action to benefit their interests irrespective of their ability to monitor or control their partners (Wu et al., 2014). In many studies, trust is considered a type of capital within social capital. Specifically, trust can be divided into two types: good faith trust and ability trust. Good faith trust refers to a high degree of integrity between partners, while ability trust is the belief that partners have sufficient resources and capabilities to meet cooperation requirements; moreover, these two types of trust can be independent of each other. If a company enjoys a high level of goodwill trust from its partners, its partners hold positive attitudes toward the corresponding alliance’s activities or have sufficient ability to achieve the alliance’s goals. That is, the more trust a business places in a partnership, the greater it will show some behavior worthy of the trust of its partner. In turn, the partner evaluates the activities of the enterprise and reacts in the same way (Hashim and Tan, 2015). Working partners in a high-trust relationship are not hesitant to share all information and trust the information they receive, which increases their willingness to partake in information sharing in return for each other’s contributions to the relationship. Therefore, trust plays a critical role in determining information sharing (Wang et al., 2014). Therefore, we propose the following:

*H2: Trust has a significant positive impact on the sustainable performance of alliances.*

### The Mediating Role of Information Sharing

Information sharing has been regarded as the most basic premise of enterprise collaboration in many studies, and even represents collaboration. Information sharing is a central process through which team members collectively utilize their available informational resources. Information sharing can be understood as the process of disseminating knowledge across individuals or organizations, thereby further integrating it into one’s own knowledge and innovating on this basis to achieve value creation. Information sharing is the basis for effective collaboration in a supply chain. Although many researchers have reported that information sharing can increase corporation performance, firms need to implement collaborative initiatives to achieve increased performance. The study of Kelle and Akbulut (2005) on the benefits of information sharing for manufacturers shows that the best performing firms not only share information with their partners but also work closely with them to achieve superior performance derived from activities such as collaborative planning and collaborative product development.

Knowledge management theory shows that a wide range of mutual exchanges, learning, and sharing is the only way to optimally use and realize the value-added effect of knowledge; moreover, the benefits of knowledge owners can be maximized in this way (Wang and Wang, 2012; Wang and Hu, 2020). Information sharing has become an integral part of organizations’ business strategies, and it aids organizations in growing, innovating in the market and gaining competitive advantages (Ganguly et al., 2019). Obtaining knowledge from other members of an alliance can enrich the knowledge reserves of members and help members carry out knowledge innovation to seize new market opportunities (Kim et al., 2011). Many studies have attempted to identify prerequisites for collaborative relationships in terms of the need for information sharing. Barratt (2004) argued that information sharing is a critical determinant of collaborative culture, as collaborative culture can effectively facilitate allied decision making among organizations. Effective information sharing among partners can be an important driver of collaborative effort and improve performance (Prajogo and Olhager, 2012). More specifically, collaboration requires that individual participants adopt e-business networks or common IT architecture to share information (Horvath, 2001). Information sharing facilitates collaborative decision making across supply chains. Firms enter networks to obtain knowledge, information, and other resources. Research shows that the content and quality of information have direct impacts on organizational practices such as manufacturing efficiency and responsiveness (Li et al., 2014).

Therefore, the following assumptions are proposed:

*H3: Alliance member information sharing mediates the effect of cooperation on the sustainable performance of alliances.*

*H4: Alliance member information sharing mediates the effect of trust on the sustainable performance of alliances.*

### The Moderating Role of Government Support

In recent years, the relationship between government support and corporate performance has attracted the attention of many scholars. To better understand whether and how government support in areas such as policies and services affects business success, scholars have conducted extensive research, and they have generally arrived at the conclusion that government support has a positive effect on businesses that is regulated by certain factors (Collewaert et al., 2010; Jugend et al., 2018; Yong et al., 2022). As a major participant of socioeconomic networks, governments’ support and intervention in enterprises greatly affects the open innovation activities of enterprises. In particular, governments have a strong driving effect on the technological innovation of enterprises in the national strategic adjustment industry; moreover, the industrial manufacturing industry, which encompasses general equipment manufacturing and professional equipment manufacturing, is particularly sensitive to changes in capital policies (Bai et al., 2019). Governments can support companies (e.g., *via* tax breaks or granting innovation funds) by alleviating the financial pressure on them to develop and innovate, and they can act as a bridge between companies and universities or the press. Governments can also help enterprises acquire information on cutting-edge technology and scientific and technological knowledge and accelerate the integration of knowledge innovation, production, education and research (Yu et al., 2016). In addition, regarding formal institutions, governments can create an appropriate institutional environment for enterprise development. In terms of financing, production and the operating environment, in relation to helping enterprises integrate internal and external resources to achieve strategic goals, the essence of government behavior is to control the macro innovation environment, which can promote technological innovation, standard formation, transfer and diffusion and create a stable, sustained, friendly and open cooperation space between enterprises (Huang et al., 2019). Government support can be seen by partners as a positive signal that can act as a “microphone” that helps enterprises obtain the external innovation resources they need and increase their input and output, thereby improving their sustainable performance (Malik and Kotabe, 2009; Han et al., 2018; Guo et al., 2020). Therefore, we propose the following:

*H5: Government support moderates the impact of member information sharing on corporate sustainability performance.*

The research model in this article is shown in Figure 1:

## Materials and Methods

This research mainly adopts the method of commissioned survey. Through the recommendation of the Sichuan Sports Association, electronic questionnaires were distributed among sports equipment manufacturers in 25 provinces, autonomous regions and municipalities affiliated to the China Sports Association. The issuance period is from December 2021 to February 2022. The questionnaire was aimed at various types of participants in a strategic alliance in the sports equipment manufacturing industry. A total of 400 electronic questionnaires were distributed, and 346 questionnaires were recovered for a recovery rate of 86.5%. The questionnaires were filtered according to the following criteria: questionnaires with many identical selected numbers and those that had numbers filled in with a certain level of regularity were considered invalid. According to the above criteria, a total of 35 invalid questionnaires were excluded, and 311 valid questionnaires were retained for an effective rate of 90%. To ensure the validity and reliability of the measurement tools, this study adopted scales previously used in the literature. And through the opinions of three professors and three industry experts, some adjustments are made according to the characteristics of the industry alliance. The reliability and validity tests of the measurement scale and the whole model were tested using SPSS 24.0 software and AMOS 24.0 software.

Table 1 gives additional information on the variable definitions.

**TABLE 1:** Variables and questions.

Variables	Questions	Factor loading	Literature source
Cooperation	Alliance members promote a culture of cooperation and exchange	0.848	van Beers and Zand, 2014; Arroyave et al., 2020
	Alliance members emphasize teamwork	0.880	
	Alliance members believe that cooperation between partners is more important than competition	0.850	
	Cooperation between alliance members enables them to resolve business problems more efficiently	0.540	
Trust	Alliance members care for each other, communicate openly and trust each other	0.721	Nyaga and Whipple, 2011; Poppo et al., 2016
	Alliance members dare to invest more money in joint research and development or learning	0.728	
	Alliance members face sudden crises together	0.637	
Information sharing (IS)	Alliance members form cross-organizational learning teams and hold regular thematic discussions to share new knowledge and new technologies they have learned	0.899	Shang et al., 2016; Weeks et al., 2017; Wang et al., 2020
	Alliance members share technical knowledge with knowledge alliance partners in a timely manner through knowledge alliances	0.869	
	Alliance members actively seek to participate in the training provided by the knowledge alliance	0.830	
Government support (GS)	Local governments have enacted laws and regulations to support the development of knowledge-based enterprises and organizations	0.766	Lu et al., 2014; Ohta et al., 2021
	The government helps link knowledge partners	0.836	
	The government funds the organization of enterprises, scientific research institutes and institutions of higher learning to cooperate in basic research	0.683	
Sustainable performance (SP)	Economic performance: Your business is able to maintain a high level of profit over a long period of time	0.853	Griffiths and Finlay, 2004; Helfat and Peteraf, 2009; Ahmad, 2015
	Social performance: Your business is able to provide customers with products that satisfy them, maintaining high customer satisfaction	0.812	
	Environmental performance: Your business has strong dynamic sustainability and environmental resources	0.751	

As shown in Table 2, the correlation between the main variables in this study reached a significant level, which laid the foundation for further hypothesis testing. Moreover, the Cronbach’s α reliability coefficient values of all the scales were greater than 0.7, meeting statistical requirements.

**TABLE 2:** Mean, standard deviation, correlation analysis, and reliability test results for each variable.

		Mean	Standard deviation	1	2	3	4	5
1	Cooperation	4.00	0.79	(0.730)				
2	Trust	3.59	0.82	0.334**	(0.709)			
3	Information sharing	3.83	0.95	0.336**	0.270**	(0.705)		
4	Government support	4.05	0.84	0.124*	0.177**	0.112*	(0.745)	
5	Sustainable performance	4.26	0.76	0.291**	0.192**	0.257**	0.307**	(0.712)

**p < 0.01 and *p < 0.05; the Cronbach’s α reliability coefficients for each scale are on the diagonal in parentheses.

In an SEM, common method bias (CMB) is a phenomenon caused by an incorrect measurement method design (Fuller et al., 2016). CMB can lead to artificial variations in the relationships between variables (Malhotra et al., 2017), as the data collected do not accurately reflect the actual opinions of the sample individuals surveyed. To prevent this bias, the questionnaire was drafted following the suggestions of Podsakoff et al. (2012). Additionally, a collinearity test based on variance inflation factors (VIFs) was performed to detect the presence of CMB (Kock, 2015). A VIF above 3.3 would indicate the existence of collinearity and thus that the model may be affected by CMB. The model did not include any VIFs greater than 2.4 and could thus be considered free of CMB (Valero-Amaro et al., 2021).

## Results and Discussion

In this article, multiple regression models were used to examine the main effects, mediating effects, and regulatory effects.

### Main Effects Test

Using linear regression, the impacts of the two dimensions of alliance partnership, namely, cooperation and trust, on the sustainable performance of alliances were examined separately, and the linear regression test results are presented in Table 3. With controls for gender, age, and experience, the macro plug-in PROCESS3.3 of SPSS24.0 was used to test the research hypothesis.

**TABLE 3:** Regression analysis of the impact of alliance partnerships on alliance sustainability performance.

Independent variable		Dependent variable: Alliance sustainability performance		
	
	M1	M2	M3	M4
Gender	–0.026	–0.015	–0.025	–0.016
Age	0.013	0.085	0.053	0.101
Experience	0.134*	0.134*	0.154**	0.147**
Cooperation		0.315**		0.272**
Trust			0.222**	0.139*
*R* ^2^	0.020	0.113	0.067	0.130
Δ*R*^2^	0.020	0.094	0.047	0.110
Δ*F*	2.067	32.33**	15.34**	19.36**

N = 311; **p < 0.01 and *p < 0.05; two-tailed test.

As shown in Table 3, in both M2 and M3, cooperation and trust have significant positive impacts on sustainable performance (β = 0.315, *p* < 0.01; β = 0.222, *p* < 0.01). Therefore, the study hypotheses H1 and H2 were verified. When cooperation and trust were both included in the regression models, their impact on alliance sustainability performance remained significant (M4, β = 0.272, *p* < 0.01; β = 0.139, *p* < 0.01).

### Mediation Effect Test

Using the multiple regression analysis method, we tested the mediating role of alliance information sharing in the impacts of the two dimensions of alliance membership, namely, cooperation and trust, on sustainable alliance performance. Table 4 presents the linear regression test results. After incorporating the control variables, cooperation, trust, and information sharing were incorporated into the regression model.

**TABLE 4:** Analysis of the mediating effect of information sharing.

Independent variable	Dependent variable: Information sharing		Dependent variable: Alliance sustainability performance			
	M1	M2	M3	M4	M5	M6
Gender	–0.033	–0.022	–0.015	–0.011	–0.025	–0.018
Age	−0.190**	–0.100	0.085	0.108	0.053	0.086
Experience	0.047	0.063	0.134*	0.125*	0.154**	0.139*
Cooperation		0.257**	0.315**	0.256**		
Trust		0.172**			0.222**	0.165**
Information sharing				0.188**		0.225**
*R* ^2^	0.036	0.153	0.113	0.144	0.067	0.112
Δ*R*^2^	0.036	0.116	0.094	0.124	0.047	0.093
Δ*F*	3.87*	20.97**	32.33**	22.18**	15.34**	15.89**

N = 311; **p < 0.01 and *p < 0.05; two-tailed test.

As shown in Table 4, in M2, cooperation and trust were shown to have significant positive impacts on the information sharing of alliance members (β = 0, *p* < 0.05; β = 0, *p* < 0.01). In both M3 and M5, cooperation and trust have significant positive impacts on the sustainability performance of the alliance (β = 0.315, *p* < 0.01; β = 0.222, *p* < 0.01). With the inclusion of alliance member information sharing in the regression model, the impacts of cooperation and trust on sustainable performance remained significant but declined (M4, β = 0.256, *p* < 0.01; M10, β = 0.165, *p* < 0.01). Moreover, the sharing of information among alliance members was shown to have a significant impact on sustainable performance (β = 0.188, *p* < 0.01; β = 0.225, *p* < 0.01). This shows that the sharing of information among alliance members plays a mediating role in the impacts of cooperation and trust on sustainable performance. Therefore, hypotheses H3 and H4 were verified.

The deviation correction non-parametric percentile bootstrapping method was used to repeatedly sample 10,000 times to facilitate a mediation effect test, and the results are shown in Table 5.

**TABLE 5:** Bootstrapping analysis of the mediating effect of information sharing.

Model	Direct effects P_YX_	95% BC CI	Indirect effects P_YM_P_MX_	95% BC CI	Total effect P_YX_ + P_YM_P_MX_	95% BC CI
Cooperation→IS→SP	0.341*	[0.031,0.626]	0.098*	[0.004,0.231]	0.439**	[0.151,0.691]
Trust→IS→SP	0.040	[−0.179,0.248]	0.029	[−0.034,0.099]	0.069	[−0.160,0.271]

** and * indicate significant correlations at the p < 0.001, 0.01, and 0.05 levels (double tailed), respectively. P_YX_ represents the effect of the anterior dependent variable on the result variable; P_MX_ represents the influence of the antecedent variable on the mediation variable; and P_YM_ stands for the effect of a mediation variable on a result variable.

As shown in Table 5, the value corresponding to the mediating effect of information sharing among alliance members on the relationship between cooperation and sustainable performance is 0.098, 95% BC CI = [0.004,0.231], 0 is not included in the interval, and the mediating effect is significant. The value corresponding to the mediating effect of alliance member information sharing on the relationship between trust and sustainable performance is 0.029, 95% BC CI = [−0.034,0.099], and the mediating effect is not significant.

### Moderating Role of Government Support

Linear regression was used to test the moderating role of government support in the impact of the information sharing of alliance members on sustainable performance. Table 6 presents the linear regression test results.

**TABLE 6:** Moderating effect of government support.

Independent variable		Dependent variable: Sustainability performance		
	
	M1	M2	M3	M4
Gender	–0.026	–0.018	–0.037	–0.028
Age	0.013	0.063	0.055	0.051
Experience	0.134*	0.121*	0.088	0.079
Information sharing		0.266**	0.234**	0.229**
Government support			0.271**	0.237**
IS × GS				−0.231**
*R* ^2^	0.020	0.088	0.159	0.211
Δ*R*^2^	0.020	0.068	0.071	0.052
Δ*F*	2.07	22.83**	25.79**	20.07**

N = 311; **p < 0.01 and *p < 0.05; two-tailed test.

Table 6 shows that the interaction items of information sharing and government support in M4 have a significant negative impact on sustainable performance (β = −0.231, *p* < 0.05); therefore, research hypothesis H5 is verified.

**FIGURE 2:**
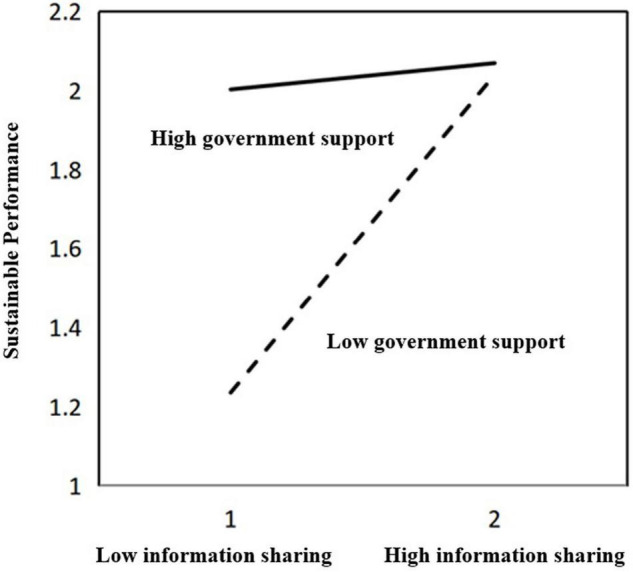
The moderating effect of government support.

Next, according to the suggestion of Aiken et al. (1991), a schematic diagram of the moderating effect of government support on the relationship between information sharing and sustainable performance was made based on a simple slope analysis point method. We constructed high- and low-level moderator variables representing government support for this regression analysis. Figure 2 and the regression analysis show that when government support is high, the positive effect of member information sharing on sustainable performance is relatively weak (β = 0.034, n.s.). When government support is low, the positive effect of member information sharing on sustainable performance is significantly enhanced (β = 0.423, *p* < 0.01).

## Conclusion

### Research Conclusion

As important participants of emerging markets, manufacturing companies not only follow the inherent requirements of green economic development but also assume responsibility for actively responding to environmental challenges. In the context of the knowledge economy, to prevent the uncertainty caused by competition between related enterprises in industries, enterprises are giving increasing attention to obtaining new knowledge from outside their industries. Therefore, industrial alliances have become the main structural form and source that enterprises use realize sustainable development. Based on previous research, this study summarizes the influencing factors of partnership, information sharing, and government support, constructs a conceptual model of the mechanism of action between these variables and the sustainable performance of an industry, and proposes the basic assumptions of the research. To test these hypotheses, a questionnaire was designed that was used to obtain relevant data by means of a scale, and these data were statistically analyzed based on correlation analysis. The reliability and validity tests of the measurement scale and the whole model were tested using SPSS 24.0 software and AMOS 24.0 software. Through theoretical analysis and empirical research, the following conclusions are obtained. (1) The positive impact of partnership and member information sharing on the sustainable performance of industry alliances is verified. (2) The mediating effect of member information sharing in partnership on the sustainable performance of industry alliance is verified. (3) It verifies the moderating effect of government support in the impact of information sharing on sustainable performance.

### Theoretical Contribution

This research has practical guiding significance for improving the theory of alliances and for enterprises and organizations aiming to enhance their green development and sustainable development through alliances.

(1) Partnerships and member information sharing have significant positive impacts on the sustainable performance of industry alliances.

Partnerships are mainly characterized by cooperation and trust among members. The cooperation and trust of members are the basis for further collaboration. The theoretical assumptions that motivation induces behavior and that willingness guides action are also applicable from the study of individual behavior to organizational behavior. Information sharing is the foundation of and key link to promoting knowledge development. An industry alliance is not an enterprise organization based on equity but a loose alliance based on knowledge development.

(2) Information sharing plays a partial mediating role in the impact of partnerships on the sustainable performance of industry alliances.

Information sharing is used not only to transmit information to another party but also to digest and absorb shared knowledge, integrate it existing knowledge structures, and develop new knowledge capabilities. In the era of big data, enterprise project teams should strive to realize the complementarity of potential absorptive capacity and actual absorptive capacity, especially to strengthen the acquisition and learning of external information. Only in this way can the potential absorptive capacity of an enterprise be transformed into its innovation achievement and sustainable development be achieved.

(3) Government support plays a moderating role in the impact of information sharing on sustainable performance.

The government can not only formulate policies to support the green development of industries but also directly provide resources. In addition, the government can provide industry alliances with public infrastructure and resources, green and sustainable development subsidies, science and technology funds, etc., to support such alliances in increasing their green innovation activities and enhancing their competitiveness and sustainable development level.

### Limitations and Future Research Directions

Due to a lack of research experience and resource constraints, this study is still insufficient. First, most of the questionnaires used in this study were developed based on foreign national conditions; thus, it is uncertain whether they are suitable for the Chinese cultural situation. Therefore, in future research, we can further develop a questionnaire suitable for the local cultural situation of China. Second, in terms of data collection, the measurement of the core variables used in this study was based on employee self-assessment and carried out during a single period, so it is difficult to further clarify the causal relationship of the model. In future research, a time series design and other evaluation methods will be used to collect data to reduce CMB.

## Data Availability Statement

The datasets presented in this study can be found in online repositories. The names of the repository/repositories and accession number(s) can be found in the article/supplementary material.

## Author Contributions

LY designed the study and analyzed the data. QC discussed the results. MY drafted the manuscript. Finally, the authors have agreed to be accountable for all aspects of the manuscript in ensuring that questions related to the accuracy or integrity of any part of it are appropriately investigated and resolved. All authors contributed to the article and approved the submitted version.

## Conflict of Interest

The authors declare that the research was conducted in the absence of any commercial or financial relationships that could be construed as a potential conflict of interest.

## Publisher’s Note

All claims expressed in this article are solely those of the authors and do not necessarily represent those of their affiliated organizations, or those of the publisher, the editors and the reviewers. Any product that may be evaluated in this article, or claim that may be made by its manufacturer, is not guaranteed or endorsed by the publisher.
